# Development of PBC/SSc overlap syndrome in chronic GVHD patient: immunological implications in the presence of mitochondrial, nucleolar and spindle midzone autoantigens 

**Published:** 2017

**Authors:** Roberto Assandri, Federico Serana, Alessandro Montanelli

**Affiliations:** 1 *Clinical Investigation Laboratory, ASST Crema, Crema, Cremona, Italy*; 2 *Clinical Investigation Laboratory, Diagnostics Department, Spedali Civili of Brescia, Brescia, Italy*

**Keywords:** GVHD, Immunological implications, Treatment

## Abstract

Chronic Graft versus Host Disease (cGVHD) is a complex disease resulting from donor T-cell recognition of a genetically disparate recipient that is unable to reject donor cells after allogeneic Stem Cell Transplantation (HSCT). cGVHD has some features resembling to autoimmune diseases (AD) such as Sjögren syndrome, primary biliary cirrhosis (PBC) and scleroderma (SSc). Also patients with cGVHD could develop extensive cGVHD with scleroderma-like skin manifestations and other clinical signs similar to those of patients with scleroderma. We take into consideration a patient with GVHD that developed PBC/SSc overlap syndrome with a complex and particular autoantibodies profile. Indirect immunofluorescence (IIF) with double coloration showed a cytoplasmic mitochondrial-like pattern, a clumpy nucleolar staining pattern, and a cell-cycle related staining pattern. Following anaphase onset, proteins regulator of cytokinesis localizes to the overlap zone on the ends of midzone microtubules and becomes compacted during furrow ingression to form the midbody. Second level tests confirmed the presence of anti-mitochondrial antibodies M2-subunit but no other autoantibodies were found. We performed a home-made immunoblot analysis that identified a 37 kDa fibrillarin band, and not identify 47 kDa, 31KDa and 18/20 kDa bands. After literature review of these possible cellular localizations, the proteins recognized by our patient’s serum seem likely to be Aab to core midzone organizer components. However, due to the unavailability of the proper techniques in our laboratory, we were not able to further characterize them. The pathogenesis and morbidity of cGVHD after HSCT remains enigmatic, but the presence of specific autoantibodies are the hallmark of AD and represent a possibility of differential diagnosis. Standard techniques combined with the use of non-routinely laboratory techniques are a usefully and complementary method for studying difficult and particular cases. In fact, these autoantibodies will be considered as ‘‘diagnostic’’ and not as ‘‘esoteric’’ antibodies. In conclusion, a re-assessment of the diagnostic protocols in cGVHD together with a precise observation of the clinical and laboratory picture will ultimately help us clarify the disease and could provide a better understanding of the immune network deregulation.

## Introduction

 Allogenic Haematopoietic Stem-Cell Transplantation (HSCT) is a medical therapy for haematological malignancies and disorders of blood cells. HSCT has a major impact on the immune system, resulting in immunologic reaction by the donor lymphocytes against the recipient (1). In fact, mature T cells contained in the allografts reconstitute T-cell immunity but can also attack and eradicate malignant cells in the recipient patient (1). These T cells recognize the recipient as 'non-self' and trigger a variety of immune-mediate mechanisms that directly hit the host tissues, an event known as graft-versus-host disease (GVHD) (2). GVHD is also the major cause of late morbidity and mortality after allogenic HSCT. The chronic form of GVHD (cGVHD) is a multi-organ pathological condition, distinguished in limited and extensive, characterized by skin manifestations and/or hepatic dysfunction including involvement of other organs (2). In contrast to acute GVHD, the underlying mechanisms of cGVHD are not fully understood. For example, in the liver there is some evidence that donor T follicular helper cells play a role by causing aberrant B-cell function in germinal centers and alloantibody deposition (3). A distinctive feature of cGVHD is that many of its clinical and molecular manifestations resemble those of an autoimmune disease (AD), which is commonly defined as a self-directed inflammatory condition occurring in various tissues and organs, involving both the innate and adaptive immune system, and characterized by the production of several autoantibodies (aAbs). 

Both cGVHD and AD are characterized by the dysregulation of immune responses resulting in tissue inflammation, damage, scarring and organ dysfunction. Moreover, both conditions are probably associated with a genetic predisposition. Among AD, systemic sclerosis (SSc) is a multi-systemic condition that mainly affects the skin, lungs, gastrointestinal tract and other organs (4) leading to a severe and progressive fibrosis. In cGVHD, skin lesions resemble those of SSc. Indeed, cGVHD patients develop extensive skin scleroderma-like lesions and other SSc signs and symptoms, but above all they can present with two of the SSc hallmarks: the Raynaud phenomenon and autoantibodies (2). Primary biliary cirrhosis (PBC) is another AD characterized by autoimmune biliary epithelial cell destruction that leads to a chronic cholestatic liver disease, and shares clinical features with cGVHD. Here, we describe the case of a patient with cGVHD who developed systemic sclerosis (SSc)/ primary biliary cirrhosis overlap syndrome with a complex and particular autoantibodies profile. 

## Case report

A 59-year-old woman visited our hospital after 2 years and 8 months of HSCT by voluntary donor 9/10 match (female, HLC-C mismatch), preceded by reduced intensity conditioning regimen for non-Hodgkin mantle-cell lymphoma. 

Clinical information was obtained through a structured review of the medical records and laboratory tests

***Clinical status before transplantation***

Risks linked with immunosuppressant and chemotherapy agents were estimated with clinical and laboratory parameters. Hepatic fungal infections were evaluated using high resolution CT and fungal biomarkers (galactomannan and glucan assays). Patient received voriconazole to prevent liver infections. Viral serologies (hepatitis B surface antigen, anti - hepatitis B surface antigen, immunoglobulin G, Anti -hepatitis B core antigen, anti -hepatitis C virus, cytomegalovirus, Epstein-Barr virus, herpes simplex virus, and human immunodeficiency virus) were all negative. 

The baseline laboratory tests reported: albumin 2.9 g/dL, total bilirubin 2.1 mg/dL, AST 282 U/L, ALT 250 U/L, and GGT 151 U/L. The total protein was 63.2 g/L. Before transplantation fibrosis score was F1 (= portal fibrosis without septa,). 

***Clinical status after transplantation***

At presentation, in October 2015, mild cGVHD (oral mucositis) was observed, while the patient complained of pain in her knees, shoulders, and metatarsophalangeal joints of both hands. 

**Figure 1:**
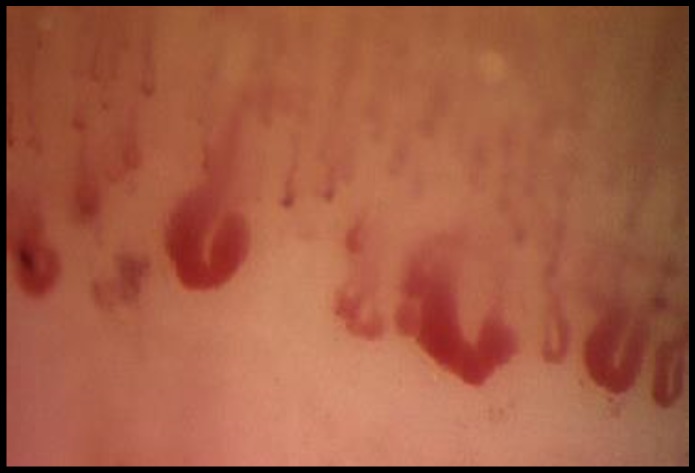
Nail-fold capillaroscopy: SSc pattern

Ten days later the patient was hospitalized. On examination she presented with sclerodactyly and digital edema, characteristic of scleroderma; she was afebrile with a blood pressure of 135/85 mm Hg and a heart rate of 76 per minute. cGVHD activity was defined with the criteria showed in Material and Methods. Nail-fold capillaroscopy findings showed giant capillaries, microhemorrhages and avascular areas typical of SSc (see Figure 1). 

**Figure 2:**
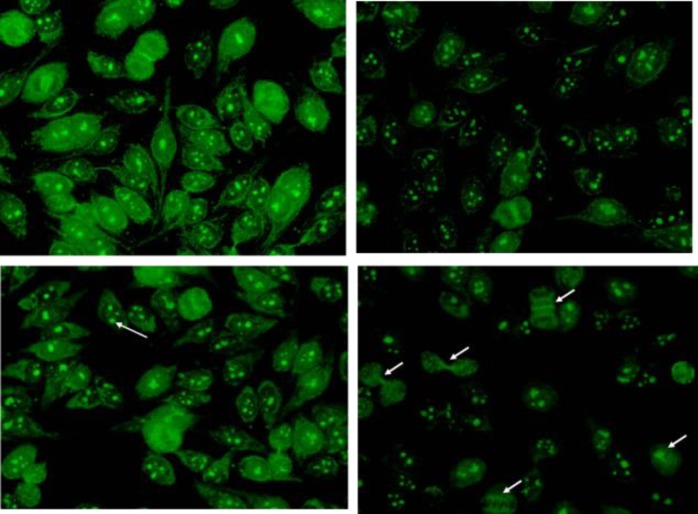
indirect immunofluorescence (IIF) on standard Hep-2000 cells patient serum

**Figure 3:**
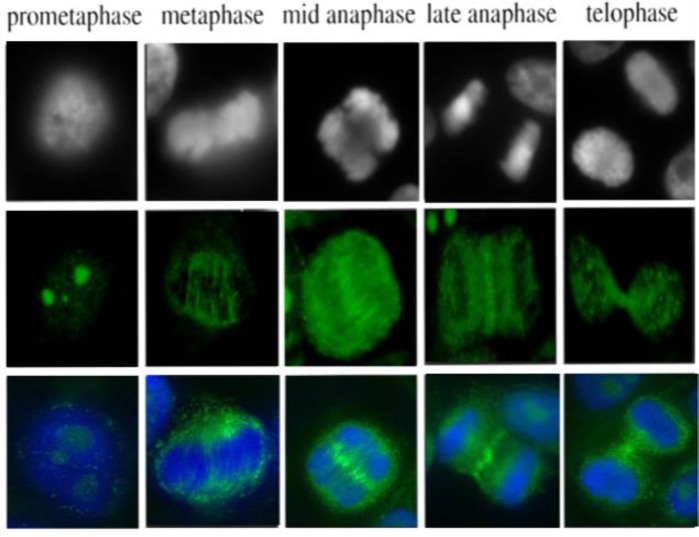
The expression and intracellular distribution of auto-antigens were followed throughout the cell cycle using immunofluorescence microscopy on Hep-2000 cell DAPI and FITC double coloration indirect immunofluorescence (IIF) revealed a multi-morphic and heterogeneous pattern. In metaphase cell antibodies decorating the spindle fibers themselves of the whole spindle fiber apparatus from pole to the chromatin plate. Following anaphase onset, proteins and related Aab localizes to the overlap zone on the ends of midzone microtubules and becomes compacted during furrow ingression to form the midbody. In telophase cells the staining is restricted to the cleavage furrow and the narrow connecting midbody between cells that are completing cytokinesis

Schirmer's tear test performed in each eye with anesthesia scored from 0-30 mm and were considered negative. Axial CT showed emphysema involving the upper lobes. No pleural effusions were observed. The alveolar capillary diffusion of carbon dioxide, performed with the single breath method, showed a mild with a lung diffusion capacity of 87%. In the same month, the patient reported widespread pain and muscle cramps in arms and legs. EMG were performed and were considered normal. High resolution CT was performed and there was no evidence of interstitial pulmonary fibrosis. 

Esophageal dysmotility was present and abdominal ultrasonography did not reveal liver cirrhosis nor splenomegaly or ascites. Figure showed cytolitic and cholestatic laboratory parameters during a follow up of 12 months. Fibrosis score were evaluated as F2. Complement C3, C4 fractions, cholestatic and cytolysis markers were evaluated during one year before hospitalization as shown in Supplementary Figure 1. In the light of this data, disease activity was considered moderate/severe. 

***Laboratory evaluation***

*Cellular distribution of the autoantigens*

The expression and intracellular distribution of auto-antigens were followed throughout the cell cycle using immunofluorescence microscopy on Hep-2000 cells. 

In a routine clinical test by indirect immunofluorescence (IIF) on standard Hep-2000 cells, serum showed a cytoplasmic mitochondrial pattern, a clumpy nucleolar staining pattern, and a cell-cycle related staining pattern at end dilution of 1: 2560 (Figure 2 and 3).

*Mitochondrial-like cytoplasmic pattern*

**Figure 4:**
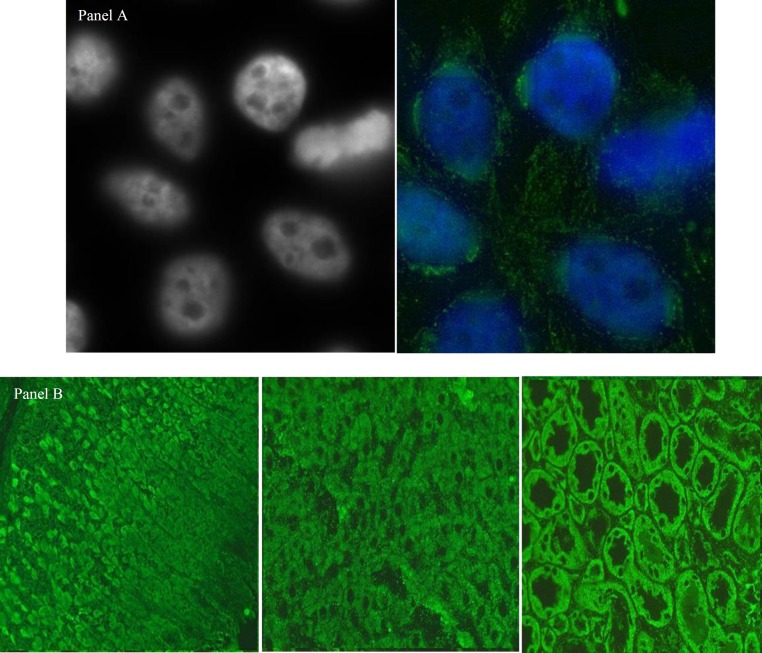
IIF- pattern were characterized by the presence of larger irregular granules extending from the nucleus throughout the cytoplasm in a reticular network. Cytoplasm of dividing cells is strongly positive (panel A). indirect immunofluorescence (IIF) on a commercial rat liver, kidney and stomach tissue with the use of polyclonal IgG, IgA and IgM antibodies confirmed the presence of AMA, with a characteristic staining pattern (Panel B

IIF- pattern were characterized by the presence of larger irregular granules extending from the nucleus throughout the cytoplasm in a reticular network. Cytoplasm of dividing cells was strongly positive (Figure 4, Panel A). IIF on a commercial rat liver, kidney and stomach tissue with the use of polyclonal IgG, IgA and IgM antibodies confirmed the presence of AMA, with a characteristic staining pattern: granular diffuse cytoplasmic staining of the Kupffer cells and hepatocytes, of the renal tubules (strongest staining is noted in distal which is mitochondria-rich) and parietal gastric cells (Figure 4, panel B). 

*Nucleolar staining pattern*

IIF Also showed diffuse fluorescence of the entire nucleolus in the interphase cells. Moreover, IIF did not show staining of chromosomes in the dense chromatin area of metaphase dividing cells (Figure 6).

*Cell-cycle related staining patter*

The expression and intracellular distribution of unknown protein were followed throughout the cell cycle using immunofluorescence microscopy as showed in Figure 7. In metaphase cell antibodies decorating the spindle fibers themselves of the whole spindle fiber apparatus from pole to the chromatin plate. Following anaphase onset, proteins and related Aab localize to the overlap zone on the ends of midzone microtubules and become compacted during furrow ingression to form the midbody. In telophase cells the staining is restricted to the the cleavage 

furrow and the narrow connecting midbody between cells that are completing cytokinesis.

***ELISA, Line immunoassay and Home-made immunoblot***

Serum were screened on ELISA (Orgentec, Mainz, Deutschland) with a mixture of extractable recombinant antigens for qualitative detection of IgG class antibodies against SS-A (Ro), SS-B (La), Sm, RNP/Sm, Scl 70 and Jo-1 in microwells. Serum tested on ELISA were considered negative (SS-A (Ro), SS-B (La), Sm, RNP/Sm, Scl 70 and Jo-1) 

Serum was tested on commercial Line immunoassays. Line immunoassay Euroline profile autoimmune liver diseases (IgG) (LIA, Euroimmun Lübeck**) ** revealed antibodies against AMA-M2. M2-E3 (BPO) but not against Sp100, PML, gp210, LKM-1, LC-1 and SLA/LPLine immunoassay Euroline profile Autoimmune diseases (IgG) (LIA, Euroimmun Lübeck) did not revealed antibodies against SS-A/Ro, SS-B/La, Sm, RNP/Sm, Scl 70 PM/Scl, CENP-B dsDNA, nucleosomes, histons, ribosomal-P protein, Jo-1 and PCNA Line immunoassay Euroline profile Systemic Sclerosis (IgG) (LIA, Euroimmun Lübeck**) **IgG class did not identify any nucleolar antibodies against proteins A and B, Scl70, RNA polymerase III, RNP Th/To, Pm/Scl, Ku and fibrillarin. Home-made blotting analysis were performed with whole cell extracts from Hep-2 culture cells. 

**Figure 5:**
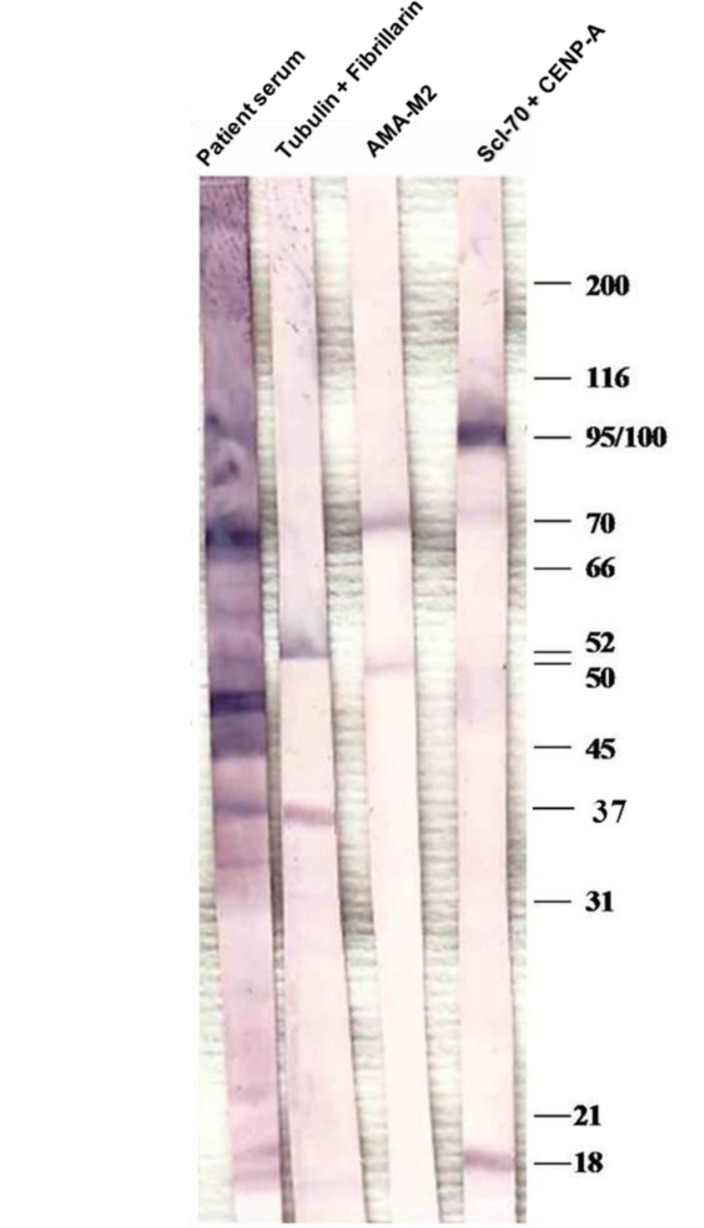
Home-made blotting analysis were performed with whole cell extracts from Hep-2. Cell extract and expressed proteins were loaded in a 10% SDS-acrylamide gel and transferred to nitrocellulose membranes in the presence of Tris-HCl-glycine-SDS buffer. Blotting was performed with the use of monoclonal alpha tubulin and fibrillarin antibody (52 and 37 KDa respectively, lane 2), AMA-M2 positive serum (lane 3) and monoclonal Topoisomerase (Scl70) and CENP-A antibodies (97 and 18 KDa respectively, Lane 4

Blotting was performed with the use of monoclonal alpha tubulin and fibrillarin antibody (52 and 37 KDa respectively, lane 2), AMA-M2 positive serum (lane 3,), monoclonal Topoisomerase (Scl70) and CENP-A antibodies (97 and 18 KDa respectively, Lane 4)

Home-made immunoblotting confirmed AMA-M2 Aab in patient serum (lane, Figure 5) whereas it revealed a 37 KDa band corresponding to fibrillarin (see Figure 5, line). Finally, immunoblotting analysis revealed non-identified 47 kDa, 31kDa and 18/20 kDa bands without correspondence to any bands in all lanes (see Figure 5).

**Figure 6:**
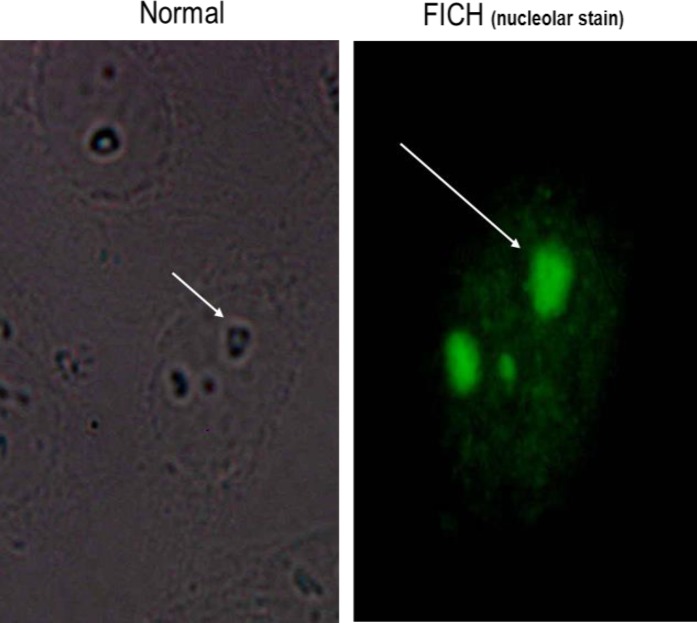
Indirect immunofluorescence (IIF) showed diffuse fluorescence of the entire nucleolus of the interphase cells and no staining of chromosomes or the “nucleolar organizing regions” in the dense chromatin area of metaphase dividing cells

## Discussion

cGVHD is a multi-organ pathological condition, distinguished in limited and extensive, characterized by skin manifestations and/or hepatic dysfunction including involvement of other organs (2). Indeed, it is now evident that the clinical manifestations of cGVHD are the result of a highly complex immune pathology involving both donor B cells and T cells, similarly to AD (4). Like in AD, during cGVHD, B cells contribute to the immune response by antibody-independent mechanisms such as antigen presentation, but also a distortion of normal B-cell homeostasis has been shown (6-8). 

**Figure 7:**
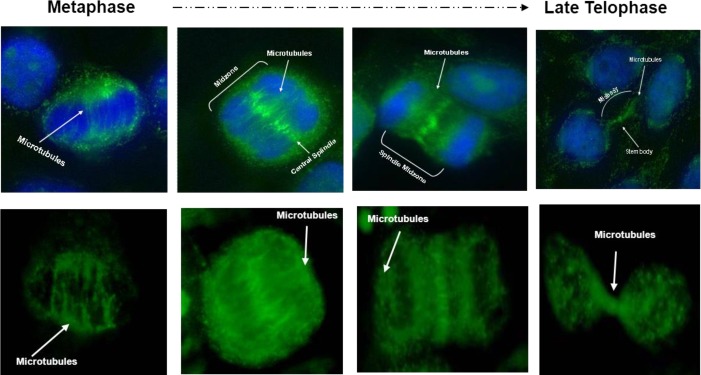
The expression and intracellular distribution of unknown protein were followed throughout the cell cycle using immunofluorescence microscopy. In metaphase cell antibodies decorating the spindle fibers themselves of the whole spindle fiber apparatus from pole to the chromatin plate. Following anaphase onset, proteins and related Aab localize to the overlap zone on the ends of midzone microtubules and become compacted during furrow ingression to form the midbody. In telophase cells the staining is restricted to the cleavage furrow and the narrow connecting midbody between cells that are completing cytokinesis

The diagnosis of cGVHD is often clinical and patients can develop extensive cGVHD with scleroderma-like skin manifestations and other clinical signs similar to those of patients with SSc. SSc, also called scleroderma, is an immune-mediated rheumatic disease that is characterized by fibrosis of the skin and internal organs and vasculopathy. Although SSc is uncommon, it has high morbidity and mortality (9). In SSc and c-GVHD, the molecular stimuli for fibrosis included similar soluble mediators, such as IL-4, IL-13 and TGF- β (10-13). Finally, as in AD, several auto-antibodies have been commonly observed in cGVHD (14), likely due to the dysregulated humoral immunity. In this condition, besides Aab that are only present in male patients receiving sex-mismatched allogeneic HSCT (15,16) and are directed against recipient Y-chromosome–encoded antigens, antibodies against non-polymorphic autoantigens can be present.

Literature also provided evidences of a SSc and cGVHD common genetic subset. Grigoryev and colleagues hypothesized that the common clinical manifestation and the common underlying players (lymphocytes) justify the combined meta-analysis of these diseases. They identified 25 gene candidates common to both cGVHD and SSc, of which primary candidates MMP2, FOSB, and DUSP1 (17). Literature provided evidences that particular antibodies specific for platelet-derived growth factor receptor have been found both in patients with SSc and cGVHD, in recent years (18,19). 

Despite these overlapping features, some clinical and laboratory features still distinguish cGVHD from SSc, like microvascular changes as evaluated by capillaroscopy and autoantibodies Raynaud’s phenomenon and nail-fold capillaroscopy changes are very characteristic in SSc but are not typical of cGVHD (20). In our patient capillaroscopy showed features suggestive of classic SSc. In 2001, Leroy and Medsger proposed a classification that takes into account the immunological profile of the patients (21). In 2013, the American College of Rheumatology/European League against Rheumatism (ACR/EULAR) criteria included a number of SSc-related antinuclear autoantibodies (ANA) such as antitopoisomerase I (Scl-70), anticentromere and anti-RNA polymerase III autoantibodies (22).

Antinuclear and anti-dsDNA, anticytoskeleton, anti-phospholipid, Anti-SSA antibodies have been described in cGVHD (14), while in sera of patients with SSc, the Aab most typically detectable are directed against DNA topoisomerase I (Scl- 70), centromeric (CENP, A-B) and nucleolar (fibrillarin, RNA polymerases, Th/To, PM/Scl) (23,24) antigens. This Aab profile seems to define an edge between the two clinical conditions. More than 90% of patients with SSc have ANA (23,24). 

In our case, serum showed a cytoplasmic mitochondrial-like pattern, a clumpy nucleolar staining pattern, and a cell-cycle related staining pattern. Anti-mitochondrial antibodies were further characterized by using a Commercial Line immunoassay revealing AMA M2 and E3 subtypes bands, whose molecular target antigens are members of 2-oxoacid dehydrogenase complex of enzymes within the mitochondrial inner membrane. Of interest, anti-mitochondrial M2 antibodies are usually a very specific finding of primary biliary cirrhosis (PBC), a disease that can be also associated with SSc (25), very rarely with autoimmune hepatitis (26). 

A clumpy nucleolar staining pattern was also observed. Commercial second level immunoblot did not identify any nucleolar auto-antigen, whereas our home-made Immunoblotting analysis revealed a 37 KDa band corresponding to fibrillarin, a basic protein that is one of the major autoantigens of the nucleolar structure, and is also over-expressed in SSc fibroblasts, indicating them as a potential autoantigen source to initiate and maintain a B cell response (23). 

Among these, of particular interest appeared Aab staining the cleavage furrow and the narrow connecting midbody between cells that were completing cytokinesis. Home-made immunoblotting analysis revealed a 47 kDa, a 31KDa and 18/20 kDa bands that did not have any correspondence to any bands in all lanes. Interestingly, in rare and particular cases, AAb to cell cycle associated antigens of the nuclear mitotic apparatus, the stem body, the cleavage furrow, and the midbody region were also found in SSc (27).

In this context, a number of molecules have been identified at the central overlap zone of the central spindle and the midbody. They can be classified into three categories. The first class includes the core microtubule organisers, such as protein regulator of cytokinesis 1 (PRC1) and centralspindlin (28). The second category is represented by mitotic kinase a component of the chromosomal passenger complex (CPC) (29). The last class included the effector of the process such as the major activator of Rho GTPase, and centrosomal protein of 55 kDa (CEP55) (30). After literature review of these possible cellular localizations, the proteins recognised by our patient’s serum seem likely to be Aab to core midzone organizer components. However, due to the unavailability of the proper techniques in our laboratory, we were not able to further characterize them.

 The results described above, do not aim to represent a standard laboratory routine procedure but reproduce the methodological approach for a complete characterization of complex antibodies profile. As a remark, in these overlapping situations it is particularly important that routinely used techniques are combined with conventional, yet home-made laboratory techniques in order to provide a successful characterization of the fine features of particular cases. (31).

The study provided an opportunity to discuss the role of traditional approach by indirect immunofluorescence investigation.

As a part of diagnostic protocols, IIF for detection the ANA remains the non-discussed screening assay. An important question is whether the IIF screening test has sufficient sensitivity to detect all clinically relevant Aab. This deficiency has led some laboratories to leave the IFI as a first step of the diagnostic tool. The ability to identify multiple Aab in a single serum is now made possible by using multiplexed diagnostic platforms. Not only some of these antibodies appear quite similar by routine IIF, but also they are often seen in association with other antibodies and in these cases, the contribution of other technologies can be useful.

In conclusion, the strong collaboration between clinician and laboratory is the only possibility for a real progress. A re-assessment of the diagnostic protocols in cGVHD together with a precise observation of the clinical and laboratory picture will ultimately help us clarify the disease and could provide a better understanding of the immune network deregulation.

## Conflict of interests

The authors declare that they have no conflict of interest.
